# Pathologic Complete Response to Modified FOLFIRINOX Enabling Conversion Surgery in Pancreatic Acinar Cell Carcinoma on Hemodialysis: A Case Report

**DOI:** 10.70352/scrj.cr.25-0827

**Published:** 2026-05-01

**Authors:** Kazuki Sekine, Yoshinori Takeda, Atsushi Takahashi, Shoichi Irie, Yoshihiro Hirata, Takaaki Kato, Ryuji Yoshioka, Yoshihito Kotera, Yoshihiro Mise, Akio Saiura

**Affiliations:** Department of Hepatobiliary-Pancreatic Surgery, Juntendo University School of Medicine, Tokyo, Japan

**Keywords:** pancreatic cancer, unresectable, locally advanced, hemodialysis, pathological complete response, acinar cell carcinoma

## Abstract

**INTRODUCTION:**

With recent advances in systemic chemotherapy for pancreatic cancer, a subset of patients with initially unresectable disease, particularly those who respond favorably to treatment, can now undergo conversion surgery (CS) and achieve prolonged survival. However, in patients undergoing hemodialysis (HD), optimal chemotherapy dosing and dialysis scheduling have not been established, raising concerns about increased chemotherapy-related toxicity and uncertain antitumor efficacy. Here, we report a case in which chemotherapy was successfully administered to a HD patient with unresectable locally advanced pancreatic cancer.

**CASE PRESENTATION:**

A 48-year-old male on maintenance HD was diagnosed with pancreatic head cancer, pathologically confirmed as acinar cell carcinoma (ACC). The tumor showed a 270° involvement of the superior mesenteric vein and a 200° contact with the superior mesenteric artery. The patient received 19 cycles of modified FOLFIRINOX (mFFX) with a uniform 40% dose reduction under an adjusted dialysis schedule. The primary tumor decreased in size, achieving a partial response according to Response Evaluation Criteria In Solid Tumors (RECIST). Subsequently, the patient underwent pancreaticoduodenectomy with portal vein resection. Pathological examination revealed a pathological complete response. No adjuvant chemotherapy was administered, and no recurrence has been observed during 3 years of postoperative follow-up.

**CONCLUSIONS:**

We successfully administered prolonged mFFX in a dialysis patient with unresectable pancreatic head ACC, enabling CS with curative intent. This case suggests that carefully dose-adjusted mFFX with appropriate dialysis scheduling can be a feasible and effective treatment option for selected HD patients with unresectable pancreatic cancer.

## Abbreviations

ACC
: acinar cell carcinoma
CS
: conversion surgery
FFX
: FOLFIRINOX
GnP
: gemcitabine + nab-PTX
HD
: hemodialysis
mFFX
: modified FOLFIRINOX
ORR
: objective response rate
pCR
: pathological complete response
plSMA
: the nerve plexus around the SMA
PR
: partial response
RECIST
: Response Evaluation Criteria In Solid Tumors
SMA
: superior mesenteric artery
SMV
: superior mesenteric vein
UR-LA
: unresectable locally advanced

## INTRODUCTION

Pancreatic cancer is diagnostically challenging and is often detected at an advanced stage; at diagnosis, UR-LA disease accounts for approximately 30%–35% and unresectable metastatic disease for about 50%.^1)^ For unresectable pancreatic cancer, systemic chemotherapy is the standard first-line treatment and often the only option. With the advent of regimens such as FFX and GnP, an increasing proportion of initially unresectable cases can proceed to CS, which has been reported to yield survival outcomes comparable to those of patients with initially resectable disease.^2,3)^

Among patients undergoing HD, cancer is one of the leading causes of death, and chemotherapy remains a standard treatment for advanced disease.^4,5)^ However, large randomized trials such as PRODIGE 24–CCTG PA.6 excluded patients on dialysis, leaving the efficacy and safety of these regimens in this population undefined.^6)^ In patients receiving HD, questions regarding optimal dosing and dialysis scheduling raise concerns about the increased chemotherapy-related toxicity and uncertain antitumor efficacy. Despite the growing number of patients on HD, no consensus has been established regarding the optimal administration of chemotherapy in this setting.^4)^

Herein, we report an HD patient with unresectable pancreatic head cancer who successfully received prolonged mFFX followed by CS.

## CASE PRESENTATION

**Fig. 1:**
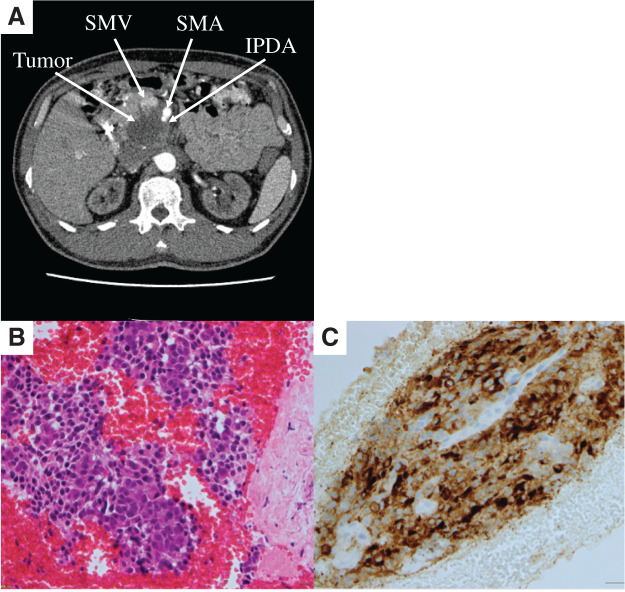
(**A**) CT findings before chemotherapy. A 56 × 47-mm tumor was observed in the pancreatic head, showing a 270° involvement of the SMV and a 200° involvement of the SMA. (**B**) Hematoxylin and eosin staining shows atypical cells with enlarged nuclei and prominent nucleoli arranged in cohesive clusters. (**C**) Immunohistochemistry shows diffuse cytoplasmic positivity for B-cell lymphoma/leukemia 10 in the tumor cells. IPDA, inferior pancreaticoduodenal artery; SMA, superior mesenteric artery; SMV, superior mesenteric vein

A 48-year-old male was referred to our department after an abdominal US performed by his primary care physician revealed a mass in the pancreatic head. His medical history included IgA nephropathy, obstructive sleep apnea, type 2 diabetes mellitus, and diabetic nephropathy. He had been undergoing HD 3 times per week. CT revealed a 56 × 47-mm tumor in the pancreatic head, with a 270° involvement of the SMV and a 200° involvement of the SMA (**Fig. 1A**). No lymph node or distant metastases were evident. Endoscopic US-guided fine-needle aspiration suggested pancreatic ACC, supported by diffuse cytoplasmic positivity for B-cell lymphoma/leukemia 10 in the tumor cells on immunohistochemistry (**Fig. 1B** and **1C**). Tumor markers were within normal limits (carcinoembryonic antigen [CEA]: 1.6 ng/mL, carbohydrate antigen 19-9 [CA19-9]: 9 U/mL, Duke pancreatic monoclonal antigen type 2 [DUPAN-2]: ≤25 U/mL). The disease was therefore classified as UR-LA pancreatic cancer, and systemic chemotherapy was initiated.

**Fig. 2:**
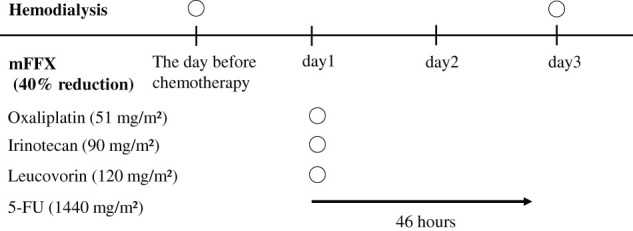
Modified FOLFIRINOX schedule and timing of hemodialysis. 5-FU, 5-fluorouracil; mFFX, modified FOLFIRINOX

**Fig. 3:**
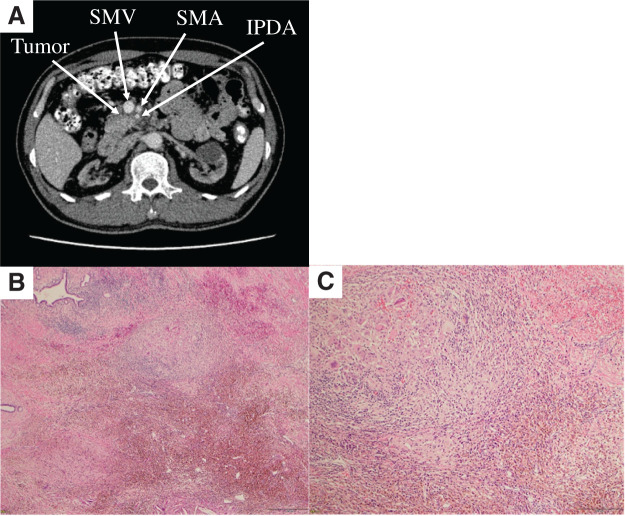
(**A**) CT findings after chemotherapy. The tumor size decreased to 15 × 13 mm, and the degree of the SMA/SMV contact was reduced to 80°. (**B**) The specimen showed necrosis with prominent surrounding fibrosis and scattered inflammatory cells. (**C**) No viable tumor cells were identified in the examined sections. IPDA, inferior pancreaticoduodenal artery; SMA, superior mesenteric artery; SMV, superior mesenteric vein

mFFX was selected for chemotherapy because the patient’s performance status was good and UGT1A1 was wild type. Because of the patient’s dialysis dependency, the doses of all agents were reduced by 40%: oxaliplatin 51 mg/m^2^, leucovorin 120 mg/m^2^, irinotecan 90 mg/m^2^, and 5-fluorouracil (5-FU) 1440 mg/m^2^ (46 hours of continuous infusion). Chemotherapy was administered every 2 weeks for 10 months. HD was performed on the day before chemotherapy and on day 3 of each cycle (**Fig. 2**). Following 19 courses (10 months) of mFFX administered without adverse events, the tumor decreased to 15 × 13 mm (RECIST, PR). The angle of contact with SMV and SMA decreased to 80° (**Fig. 3A**). Tumor marker levels remained within normal limits (CEA: 3.3 ng/mL, CA19-9: 19 U/mL, DUPAN-2: ≤25 U/mL). Accordingly, the patient proceeded to CS. Performance status and nutritional status were well maintained during chemotherapy.

After confirmation of no peritoneal dissemination or liver metastasis in staging laparoscopy, the patient underwent subtotal stomach-preserving pancreaticoduodenectomy with portal vein resection and reconstruction. Pancreatic head nerve plexus was dissected *en bloc*, while plSMA was preserved. A portion of the dissected pancreatic head nerve plexus was submitted for intraoperative frozen-section analysis and confirmed to be free of malignancy. The SMV segment suspected of invasion was resected (length 27 mm) and reconstructed by end-to-end anastomosis. Reconstruction was performed according to the Child type IIA method. The patient developed a postoperative pancreatic fistula (Clavien–Dindo grade 2, International Study Group of Pancreatic Surgery grade B) and diarrhea (Clavien–Dindo grade 2). In the early postoperative period, HD was occasionally unable to achieve the planned ultrafiltration volume because of intravascular dehydration secondary to diarrhea. However, with adequate control of diarrhea using opioid agents, HD was subsequently performed safely in most sessions. The postoperative hospital stay was 48 days because of the treatment of postoperative pancreatic fistula.

Histopathological examination revealed dense fibrosis and multinucleated giant cells at the previous tumor site, with no viable tumor cells. All dissected lymph nodes showed fibrotic changes without metastatic involvement. Based on these findings, the patient was diagnosed with a pCR (**Fig. 3B** and **3C**).

No adjuvant chemotherapy was administered because the survival benefit of adjuvant chemotherapy, including S-1, gemcitabine, and FFX, remains unclear in ACC.^6–8)^ No recurrence was observed during 3 years of postoperative follow-up.

## DISCUSSION

We successfully administered prolonged mFFX in an HD patient with UR-LA pancreatic head ACC. Despite a 40% dose reduction, pathological examination demonstrated a complete response. Following CS, the patient has remained disease-free for 3 years.

Prolonged disease control with chemotherapy is required to achieve CS.^9,10)^ Similarly, in our department, patients with UR-LA pancreatic cancer are considered eligible for CS if they have received at least 8 months of chemotherapy and have achieved sustained tumor shrinkage along with a decrease in CA19-9 levels.^9)^ However, no standardized strategy has been established for the safe and durable administration of chemotherapy in patients undergoing HD.^4)^ Among patients receiving HD, altered pharmacokinetics, immune dysfunction, and a high burden of comorbidities increase the risk of serious adverse effects. Funakoshi et al. reported that among dialysis patients with various cancers, the completion rate of perioperative chemotherapy was only about 50%, and approximately 20% of the patients died of non-cancer causes within 3 years.^4)^ Therefore, in dialysis patients, therapeutic indications should be carefully determined, considering the expected prognosis. In the present patient, mFFX was administered for 19 cycles without significant toxicity. Our case suggests that carefully dose-adjusted mFFX with appropriate dialysis scheduling can be a feasible and effective long-term treatment option for selected HD patients with unresectable pancreatic cancer. To our knowledge, this is the first report of CS in a HD patient with pancreatic cancer.

Sustaining cytotoxic chemotherapy in patients on HD depends on 2 key factors: dose intensity and HD-synchronized scheduling. First, there is no consensus on dose adjustment across agents; within mFFX, reductions are commonly recommended for oxaliplatin (30%–50%) and irinotecan (to 50 mg/m^2^ per week), whereas pharmacokinetic data do not necessarily mandate lowering the other components.^11)^ Given the small, heterogeneous evidence base, we applied a uniform 40% reduction in this patient.^12)^ This approach may have enhanced deliverability, particularly for 5-FU and leucovorin, because 5-FU metabolites such as monofluoroacetate have been implicated in hyperammonemia in HD patients.^11,12)^ Second, in contrast to dosing, recommendations on the timing of administration relative to HD are relatively concordant across pharmacokinetic studies. Janus et al. emphasized the importance of considering drug clearance during HD. For agents with high dialyzability, chemotherapy is preferably administered after HD to avoid loss of efficacy, whereas for agents with low dialyzability, the timing of administration is less critical.^13)^ Pirovano et al. also summarized the available evidence and reported recommendations that are generally consistent with this pharmacokinetic-based approach, while emphasizing that the evidence remains limited. In their review, oxaliplatin, irinotecan, and 5-FU are generally administered after HD or on non-dialysis days. In contrast, no specific recommendation has been established for the timing of leucovorin administration. Based on these considerations, we determined the timing of chemotherapy administration in the present case.^14)^ Under this combined strategy, the patient received mFFX for 10 months without serious adverse events.

On the other hand, dose reduction poses a difficult clinical dilemma because it may compromise therapeutic efficacy. Although Vary et al. found no definitive cutoff dose required to achieve disease control, previous studies have suggested that a higher relative dose intensity is associated with higher ORRs (**Table 1**).^15–26)^ Lee et al. evaluated the optimal first-line FFX dose for maintaining tumor response in 156 patients with pancreatic cancer and recommended a cumulative relative dose intensity of >70% to preserve efficacy.^27)^ In our case, all drugs were reduced to 60% of the standard dose; nevertheless, the patient achieved a pCR of the primary tumor, which may represent a novel aspect of mFFX efficacy. Consistently, Ulusakarya et al. reported that 3 of 37 patients achieved pCR after receiving a reduced mFFX dose at 63%–68% of standard intensity.^24)^ These findings suggest that the relationship between dose intensity and tumor response may be modified by the underlying tumor biology, including mutational profile, although further studies are required to clarify this relationship.

**Table 1:** Dose intensity reduction of modified FOLFIRINOX and tumor response

Author	Number of patients	Relative dose intensity (%), median [range]					ORR (%)	DCR (%)	RECIST, n (%)			
		Oxaliplatin	Irinotecan	5-FU bolus	5-FU CIV	Leucovorin			CR	PR	SD	PD
Tezuka et al. [elderly (≥65)]^15)^	75	100	83.3	0	100	100	25.3	72.0	0 (0)	19 (25.3)	35 (46.7)	21 (28.0)
Tezuka et al. [young (<65)]^15)^	76	100	83.3	0	100	100	36.8	77.6	1 (1.3)	27 (35.5)	31 (40.8)	17 (22.4)
Cavanna et al. ^16)^	32	100	75	80	100	100	36.7	56.7	0 (0)	11 (34.4)	6 (18.8)	13 (40.6)
de Jesus et al.^17)^	44	99.5 [84.0–100]	83.3 [79.7–100]	0 [0–88.7]	100 [85.1–100]	NR	31.3	81.3	2 (4.5)	8 (18.2)	16 (36.4)	6 (13.6)
Stein et al.^18)^	66	88.9	66.7	66.7	90.9	NR	27.3	92.4	0 (0)	18 (27.3)	43 (65.2)	5 (7.6)
Li et al.^19)^	62	85	75	0	100	100	32.5	60.0	0 (0)	13 (32.5)	11 (27.5)	16 (40)
Vary A^20)^	243	80.8 [23.8–102.5]	79.3 [8.5–102.1]	74.9 [0–102.5]	84.6 [23.8–102.5]	NR	20.2	74.1	5 (2.1)	44 (18.1)	131 (53.9)	63 (25.9)
Ghorani et al.^21)^	18	80.5 [53.3–100]	64.3 [43–75]	0	83.0 [65.9–100]	NR	46.7	73.3	1 (6.7)	6 (40.0)	5 (33.3)	3 (20.0)
Ozaka et al.^22)^	69	76.1	91.4	0	95.4	99.1	40.6	84.4	0 (0)	26 (37.7)	28 (40.6)	10 (14.5)
Kang et al.^23)^	42	75.0 [51.1–75.0]	75.0 [51.1–75.0]	75.0 [51.1–75.0]	75.0 [51.1–75.0]	NR	35.7	83.3	1 (2.4)	14 (33.3)	20 (47.6)	7 (16.7)
Ulusakarya et al.^24)^	37	68	63.1	63.3	63.3	NR	47.1	85.3	3 (8.1)	13 (35.1)	13 (35)	5 (14)
Yoshida et al.^25)^	31	59.6 [13.8–100]	72.8 [43.9–100]	0	77.9 [32.1–100]	82.5 [48.0–100]	38.7	74.2	0 (0)	12 (38.7)	11 (35.5)	8 (25.8)
Uemura et al. [sarcopenia]^26)^	33	54.9 [8.7–99.3]	73.1 [38.4–100]	0	82.2 [39.6-100]	88.2 [38.8–100]	12.1	78.8	0 (0)	4 (12.1)	22 (66.7)	7 (21.2)
Uemura et al. [non-sarcopenia]^26)^	36	54.4 [9.9–99.1]	77.4 [42.1–100]	0	81.7 [32.4–100]	83.3 [47.4–100]	11.1	83.3	0 (0)	4 (11.1)	26 (72.2)	6 (16.7)

Data are median [range] or number (percentage).

CIV, continuous intravenous infusion; CR, complete response; DCR, disease control rate; ORR, objective response rate; PD, progressive disease; PR, partial response; SD, stable disease

The response to chemotherapy is a key determinant in achieving CS. Because standard chemotherapy for ACC has not been established due to its rarity, we selected the preoperative chemotherapy regimen based on treatment strategies for pancreatic ductal adenocarcinoma (PDAC). mFFX has been reported to have one of the highest potentials for achieving pCR in PDAC and several studies have reported favorable outcomes in patients with ACC treated with mFFX.^28–30)^ **Table 2** shows that CS may be feasible in selected patients with locally advanced ACC or metastatic ACC who achieve a favorable response to preoperative chemotherapy. Notably, most reported cases were treated with mFFX or FFX, and these regimens appeared to be associated with relatively favorable survival outcomes compared with other treatment approaches.^31–40)^ The favorable outcome observed in patients with ACC treated with mFFX may be related, at least in part, to the unique genomic characteristics of ACC. Prior genomic studies have suggested that ACC harbors homologous recombination repair–related gene alterations, such as BRCA1/2 and PALB2, more frequently than conventional PDAC. Given that these alterations are associated with increased sensitivity to platinum-based chemotherapy, they may contribute to the favorable responses and survival outcomes observed in some ACC patients treated with mFFX or FFX.^41–43)^ In the present case, mFFX resulted in a pCR of the primary tumor, supporting that mFFX may be a promising treatment option for locally advanced ACC. Further accumulation of cases and prospective studies are warranted to validate these findings.

**Table 2:** Previous reports of conversion surgery following chemotherapy for unresectable acinar cell carcinoma

Author	Age Sex	Tumor location	Size (mm)	Metastasis and invasions	Preoperative chemotherapy	RECIST	Surgical procedure	AC	Survival (month)	Status
Distler et al.^31)^	65M	H	40	LA	5-FU, 12 courses	PR	Pancreaticoduodenectomy	None	18	NED
Yamamoto et al.^32)^	71M	H	35	Peritoneal dissemination	S-1, 1 course	PR	DP	S-1	24	NED
Endo et al.^33)^	55F	B	68	Invasion to stomach and transverse colon	GS, 4 courses	PR	DP, partial gastrectomy, and partial colectomy	GS	2	AWD
Jimbo et al.^34)^	56M	B	60	LA	FFX, 6 courses	PR	Appleby procedure	NR	NR	NR
Villano et al.^35)^	71M	T	36	Liver metastasis	FFX, 6 courses	PR	DP and partial hepatectomy	None	6	NED
Sunami et al.^36)^	37M	T	150	Liver metastasis	FFX, 12 courses	PR	DP	S-1	36	NED
Uemura et al.^37)^	67M	H	67	Invasion to omentum	FFX, 18 courses	PR	Total pancreatectomy	S-1	32	NED
Yamada et al.^38)^	60M	T	70	Liver metastasis	mFFX, 3 courses	PR	DP and extended right hemi-hepatectomy	S-1	36	NED
Watanabe et al.^39)^	70M	Bt	150	Invasion to stomach and transverse colon	1^st^ line: GnP, 1 course 2^nd^: mFFX, 6 courses	1^st^: PD 2^nd^: PR	DP, partial gastrectomy, and partial colectomy	mFFX	46	NED
Sugata et al.^40)^	67M	T	47	Liver metastasis	mFFX, 13 courses	PR	DP and partial hepatectomy	S-1	18	AWD

5-FU, 5-fluorouracil; AC, adjuvant chemotherapy; AWD, alive with disease; B, body; Bt, body and tail; DP, distal pancreatectomy; F, female; FFX, FOLFIRINOX; GS, gemcitabine + S-1; GnP, gemcitabine + nab-PTX; H, head; LA, locally advanced; M, male; mFFX, modified FOLFIRINOX; NED, no evidence of disease; NR, not reported; PD, progressive disease; PR, partial response; RECIST, Response Evaluation Criteria In Solid Tumors; T, tail

Even in patients who achieve pCR and undergo CS, the postoperative recurrence of pancreatic cancer can still occur. Stoop et al. reported that the 3-year recurrence-free survival rate in patients with pCR was approximately 70%, indicating that recurrence can still occur even after pCR.^28)^ However, in our case, adjuvant chemotherapy was not administered because the benefit of adjuvant therapy in ACC has not been established, including for regimens such as S-1, gemcitabine, and FFX.^6–8)^

The patient was young at 48 years old and had a favorable performance status and nutritional status, which may have contributed to the successful completion of long-term chemotherapy and CS. ACC is less likely to cause obstructive jaundice or pancreatitis, and thus tumor-related infections such as cholangitis or pancreatitis did not occur. This absence of infection was another important factor that enabled the patient to complete the planned chemotherapy. In addition, collaboration with the departments of gastroenterology and nephrology to determine the appropriate dosage also played a major role in the successful completion of chemotherapy without adverse events.

## CONCLUSIONS

This case suggests that the carefully dose-adjusted mFFX with appropriate dialysis scheduling may enable CS and achieve favorable oncologic outcomes in selected HD patients with initially unresectable pancreatic cancer.

## DECLARATIONS

### Availability of data and materials

All data generated or analyzed during this study are included in this published article.
